# Distributed EEG source localization of hypsarrhythmia in west syndrome: a standardized, low-resolution, brain electromagnetic tomography (sLORETA) study

**DOI:** 10.1186/s12883-025-04596-5

**Published:** 2025-12-27

**Authors:** Jooyoung Lee, Ja Un Moon, Eu Gene Park, Il Han Yoo, Ji Yoon Han, Tae-Hoon Eom, Joong Hyun Bin

**Affiliations:** Department of Pediatrics, College of Medicine, The Catholic University of Korea, Seoul, Republic of Korea

**Keywords:** West syndrome, Hypsarrhythmia, Quantitative electroencephalography (qEEG), Distributed source model, Standardized low-resolution brain electromagnetic tomography (sLORETA)

## Abstract

**Background:**

West syndrome is a developmental epileptic encephalopathy characterized by epileptic spasms, hypsarrhythmia, and neurodevelopmental regression. Despite well-defined clinical criteria, hypsarrhythmia remains challenging to characterize due to its disorganized nature. Recent advancements in quantitative electroencephalography (qEEG) provide a more objective approach to understanding the electrophysiology of this disorder. This study aims to use qEEG and distributed source localization to increase the understanding of hypsarrhythmia in West syndrome.

**Methods:**

This study involved 34 participants, 17 diagnosed with West syndrome (West syndrome group) and 17 healthy controls (control group). All participants underwent 30-minute sleep EEG recordings, and data were analyzed using standardized low-resolution brain electromagnetic tomography (sLORETA) to assess the current density distribution across four frequency bands (delta, theta, alpha, and beta). Statistical non-parametric analysis was performed to compare neural activity between the West syndrome and control groups.

**Results:**

Significant increases in current density were observed across all frequency bands in the West syndrome group, with the most pronounced difference found in the delta band, followed by theta, alpha, and beta bands. The most prominent changes in the delta band were noted in the right frontal lobe, particularly in the middle frontal gyrus. In the theta band, the most significant differences were observed in the right middle frontal gyrus, while the alpha and beta bands showed notable changes in the left inferior frontal gyrus and the right superior frontal gyrus, respectively.

**Conclusion:**

Our study demonstrates a significant increase in current density across all frequency bands, with the most pronounced differences observed in the delta band in patients with West syndrome. These changes exhibit a predominant anterior distribution, particularly within the frontal lobe. The findings provide new insights into the electrophysiology of hypsarrhythmia, highlighting the presence of a low-to-high and anteroposterior gradient in neuronal activity. These results contribute to the growing body of research on West syndrome and emphasizes the value of qEEG in enhancing the interpretation of hypsarrhythmia.

## Background

West syndrome, also known as infantile spasms, is a rare epileptic disorder predominantly affecting infants, with peak incidence observed between 4 and 7 months of age [1]. The syndrome is characterized by sudden, brief spasms or jerking movements that often occur in clusters. A key diagnostic criterion of this syndrome is the presence of hypsarrhythmia, identified by high amplitude, arrhythmic, and asynchronous slow waves, accompanied by multifocal spikes on electroencephalography (EEG), which is pathognomonic for West syndrome [2–4]. Neurodevelopmental delay or regression is also a significant aspect of this syndrome, one of the most prevalent developmental epileptic encephalopathies (DEEs) in the pediatric population [5].

The term “hypsarrhythmia” refers to a pattern of electrical activity characterized by irregularity, chaos, and disorganization [2]. Although this definition emphasizes the irregular nature of the pattern, the specific characteristics and interpretation of EEG findings lack precision. The ambiguity of the original definition, particularly regarding the degree of “irregularity,” complicates the visual interpretation of EEG [3]. Furthermore, this pattern can exhibit variations in intensity, frequency, and distribution, complicating the precise definition of hypsarrhythmia [6]. The presence of variants such as modified hypsarrhythmia further exacerbates the complexity [7]. The lack of clarity in defining hypsarrhythmia coupled with the existence of variants and subjective nature of visual interpretation can result in inter-rater variability. Consequently, the reliability of EEG-based assessments in West syndrome may be problematic, especially when treatment and prognosis decisions depend on these interpretations [3, 6, 7].

Quantitative EEG (qEEG) analysis offers a potentially more objective method for enhancing the reliability of hypsarrhythmia interpretation in the assessment of West syndrome. Several recent studies have identified the quantifiable characteristics of hypsarrhythmia using qEEG analysis, providing more knowledge regarding both West syndrome and hypsarrhythmia. These advancements have contributed to a more comprehensive understanding of the disorder [1, 4–9]. In addition, distributed models of EEG source localization for brain mapping are emerging as sophisticated tools for qEEG analysis. These models represent an advanced method for exploring the electrophysiological activity and anatomical distribution of the brain. The models offers several advantages, including the ability to identify the precise origins of abnormal electrical activity and enhance the interpretation of complex EEG patterns [10–14]. Elucidating the quantitative, spatial, and frequency characteristics of hypsarrhythmia in patients with West syndrome may be possible when using distributed models of EEG source localization.

Despite the promising potential of this approach, research regarding use in the context of West syndrome is limited. To address this deficiency, distributed models of EEG source localization were applied to examine hypsarrhythmia in West syndrome and discover novel insights into this condition. This advanced analytical method was used to obtain a more objective and reliable understanding of hypsarrhythmia in West syndrome.

## Methods

### Study population

This study included 34 participants, 17 diagnosed with West syndrome (West syndrome group) and 17 age- and sex-matched control subjects (control group). The West syndrome cohort consisted of 17 newly diagnosed patients who presented at our hospital between January 2020 and December 2024. The inclusion criteria for the West syndrome group were frequent clusters of flexor, extensor, or mixed epileptic spasms; interictal EEG results indicating hypsarrhythmia; onset age ranging from 1 to 24 months; and neurodevelopmental delay or regression subsequent to the onset of spasms [5, 15].

The associated institutional review board approved this study and written informed consent was obtained from the parents of all participants prior to data collection.

### EEG recordings and data processing

All participants underwent 30-minute sleep EEG recordings utilizing a NicoletOne™ EEG system (Natus Medical Inc., Pleasanton, CA, USA) with a sampling rate of 500 Hz. To induce sleep, chloral hydrate was administered at an initial dose of 50 mg/kg. If sedation was insufficient after 30 min, an additional 50 mg/kg dose was administered, with a maximum cumulative dosage of 100 mg/kg not exceeding 1 g. EEG recordings were started when the participants fell asleep. Twenty-one Ag/AgCl electrodes were positioned according to the international 10–20 system, covering standard temporal and parasagittal sites as well as Fz, Cz, Pz, A1, and A2. Additional electrodes were used to monitor ocular and muscle artifacts, respiration, and electrocardiography. A 19-channel EEG was recorded, and monopolar montages with a linked ear reference were utilized for data processing. Electrode impedance was maintained below 5 kΩ, with filters set at 1.0 and 70 Hz. Data were digitized online at a 16-bit resolution. Each interictal EEG recording was processed using fast Fourier transform (FFT) on 3-second epochs that were manually segmented, artifact-free, and selected from sleep stages 1–2 following visual inspection. This epoch length was considered sufficient for FFT computation and brief enough to encompass a substantial number of artifact-free segments [14, 16]. Twenty epochs were collected from each participant and were randomly selected across all interictal sleep stage 1–2 EEG recordings to ensure a representative sample. The epochs were selected blindly by one author and independently verified by another author. A total of 340 EEG samples were analyzed. The recordings were exported as American Standard Code for Information Interchange (ASCII) files and subsequently imported into standardized low-resolution brain electromagnetic tomography (sLORETA) software for further analysis.

### EEG analysis using sLORETA software

sLORETA data were generated to facilitate a comparative analysis between the two groups across four frequency bands: delta (1–4 Hz), theta (4–8 Hz), alpha (8–12 Hz), and beta (12–25 Hz). sLORETA addresses the issue of multiple testing and involves tests with all electrodes or voxels and for all time samples and discrete frequencies by executing random permutations (5,000 permutations in this study), obviating the need for additional correction for multiple comparisons [11, 17]. sLORETA, a functional imaging method grounded in electrophysiological and neuroanatomical principles, models the cortex as a series of volume elements (6,239 voxels, each measuring 5 × 5 × 5 mm). This technique is confined to the cortical gray matter, hippocampus, and amygdala and utilizes digitized Montreal Neurological Institute (MNI) coordinates adjusted to Talairach coordinates. Neuronal activity is calculated as the current density (µA/mm^2^) without assuming a specific number of active sources [11, 18]. Current density represents the strength of neuronal electrical activity spatially localized within the brain. While conceptually related to power—calculated as amplitude squared in traditional spectral analysis—current density and power differ in that current density provides a spatially resolved estimate of neuronal activity within the brain, whereas power reflects the strength of EEG signals recorded from the scalp at specific frequencies. Higher values in either measure indicate stronger synchronized neuronal firing, but their calculation domains are distinct: current density estimates the intensity of neural activity at specific brain locations, whereas power quantifies the intensity of activity measured at the scalp. The scalp electrode coordinates on the MNI brain were obtained from the International 5% system [19]. The sLORETA algorithm resolves the inverse problem by assuming that the orientations and strengths of neighboring neuronal sources (represented by adjacent voxels) are interrelated. sLORETA has been shown to be an effective tool for functional mapping due to its physiological consistency and precise localization capabilities [11]. Furthermore, the localization accuracy of sLORETA has been independently validated [20, 21]. The current density distributions in the two groups were analyzed using a voxel-by-voxel comparison of the sLORETA data for four frequency bands (delta, 1–4 Hz; theta, 4–8 Hz; alpha, 8–12 Hz; beta, 12–25 Hz). Statistical non-parametric mapping (SnPM) of sLORETA images was conducted for each contrast using built-in voxel-wise randomization tests (5,000 permutations) and a log-*F*-ratio statistic for dependent groups with thresholds of *P* < 0.01 and *P* < 0.05, corrected for multiple comparisons. Correction for multiple comparisons in SnPM with random permutations (5,000 in this study) has been shown to produce results comparable to those obtained from statistical parametric mapping using a general linear model with multiple comparison corrections derived from random field theory [17, 22].

## Results

### Baseline Characteristics

Seventeen patients with West syndrome were included in the study. Among them, 10 were classified as symptomatic: 8 had a history of brain injury, 1 had tuberous sclerosis complex, and 1 had a congenital malformation. The remaining 7 patients were classified as cryptogenic or of unknown etiology. Detailed baseline characteristics, including age, sex distribution, etiology, clinical features, and developmental status, are summarized in Table 1.

**Table 1:** Baseline characteristics of the West syndrome group

Characteristics	*n* = 17
Sex (male/female)	9/8
Age at onset of spasms (months)	7.89 ± 2.48 (mean ± SD)
< 6	10
6–12	6
12–18	1
Age at diagnosis (months)	9.35 ± 2.10 (mean ± SD)
< 6	6
6–12	9
12–18	2
Birth history	
non-specific	9
abnormal	8
Brain magnetic resonance image	
non-specific	7
abnormal	10
Developmental delay	
yes	17
no	0
Etiology	
Brain injury	8
Tuberous sclerosis complex	1
Congenital malformation	1
Cryptogenic or unknown	7

Values are presented as number (n) or mean ± SD. *SD* standard deviation

The control group consisted of 17 age- and sex-matched healthy individuals. All controls were screened for seizure-like movements, and EEG recordings confirmed normal results without evidence of epilepsy. The sex distribution was identical in both groups (9 males and 8 females each). The mean age was 9.35 ± 2.10 months in the West syndrome group and 9.02 ± 2.05 months in the control group. No significant differences were observed between the groups (*P* = 0.45).

sLORETA analysis.

**Fig. 1:**
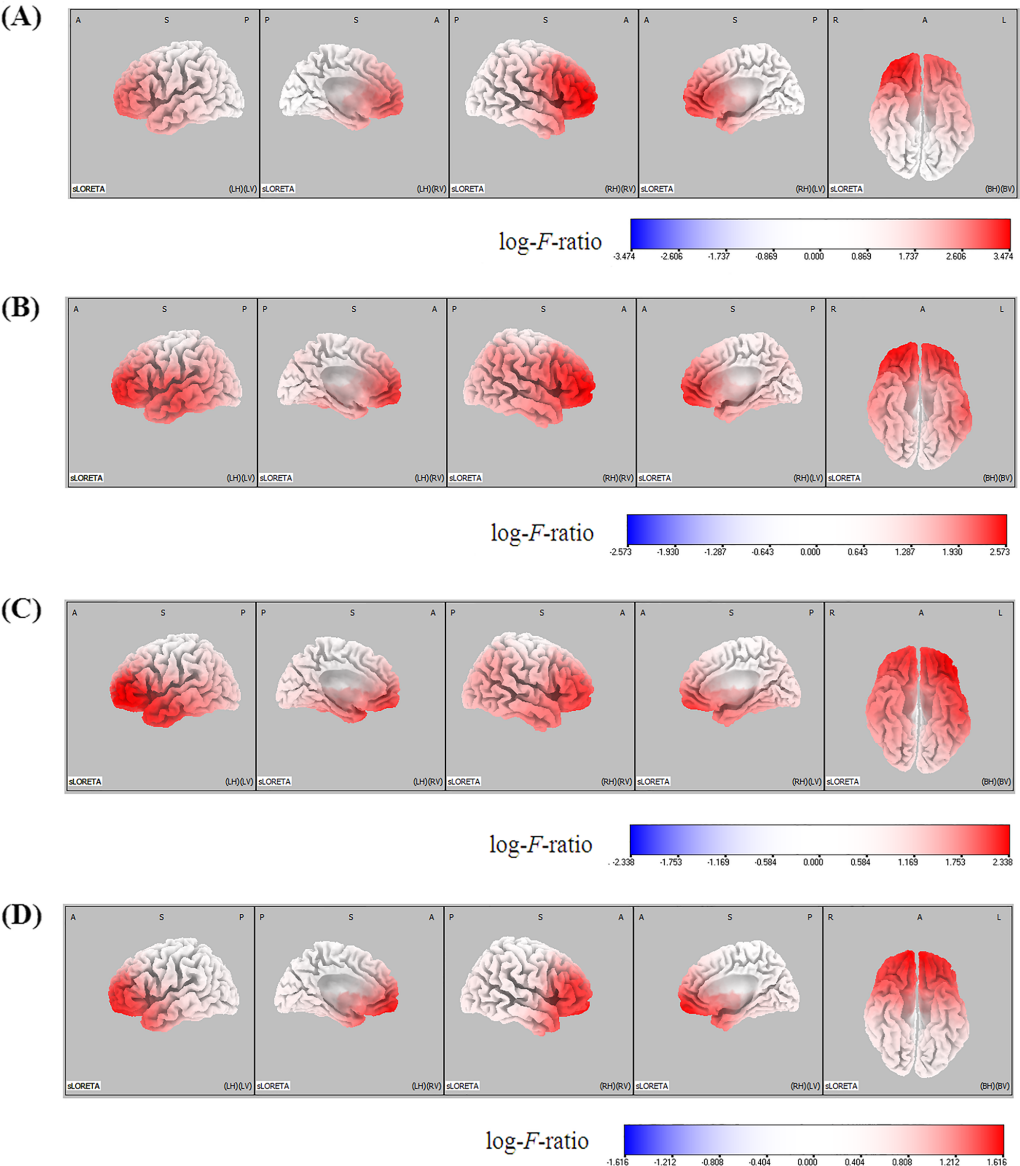
Statistical non-parametric mapping (SnPM) of standardized low-resolution brain electromagnetic tomography (sLORETA) images was conducted across four frequency bands in the West syndrome group, with comparisons made to the control group. The frequency bands analyzed included delta (1–4 Hz) **A** theta (4–8 Hz) **B** alpha (8–12 Hz) **C** and beta (12–25) Hz **D** The analysis was based on log-*F*-ratio values (threshold log-*F*-ratio = ± 0.403, *P* < 0.05; threshold log-*F*-ratio = ± 0.484, *P* < 0.01). The results were displayed on a three-dimensional fiducial brain cortex. A indicates anterior; *P* posterior, *S* superior, *I* inferior, *L* left, *R* right, *B* both, *H* hemisphere, *V* ventricle

The electrical neuronal activity across all four frequency bands was significantly elevated in the West syndrome group compared with the control group (threshold log-*F*-ratio = ± 0.403, *P* < 0.05; threshold log-*F*-ratio = ± 0.484, *P* < 0.01). Comparative analysis using SnPM of the sLORETA data revealed significant differences in current density across all frequency bands throughout the cortex, although to varying extents. Figure 1 shows the statistical maps illustrating the spatial distribution of voxels within regions that exhibited significant differences in current density on the three-dimensional fiducial cortical surface. Table 2 is a summary of the locations with the largest (top five) differences in current density with the top five locations for each frequency band.

**Table 2:** Locations of the largest (top five) differences in current density associated with background EEG activity in the two groups (West syndrome group vs. control group)

	Hemisphere	Lobe	Log-F-ratio	*P*-value
*Delta frequency band*				
Middle frontal gyrus	Right	Frontal	3.474	< 0.01
Superior frontal gyrus	Right	Frontal	3.472	< 0.01
Inferior frontal gyrus	Right	Frontal	3.465	< 0.01
Anterior cingulate	Right	Limbic	3.413	< 0.01
Insula	Right	Sub-lobar	3.376	< 0.01
*Theta frequency band*				
Middle frontal gyrus	Right	Frontal	2.573	< 0.01
Superior frontal gyrus	Right	Frontal	2.572	< 0.01
Inferior frontal gyrus	Right	Frontal	2.562	< 0.01
Medial frontal gyrus	Right	Frontal	2.523	< 0.01
Anterior cingulate	Right	Limbic	2.479	< 0.01
*Alpha frequency band*				
Inferior frontal gyrus	Left	Frontal	2.338	< 0.01
Middle frontal gyrus	Left	Frontal	2.336	< 0.01
Sub-gyral	Left	Frontal	2.327	< 0.01
Superior frontal gyrus	Left	Frontal	2.306	< 0.01
Superior temporal gyrus	Left	Temporal	2.283	< 0.01
*Beta frequency band*				
Superior frontal gyrus	Right	Frontal	1.616	< 0.01
Medial frontal gyrus	Right	Frontal	1.610	< 0.01
Rectal gyrus	Right	Frontal	1.602	< 0.01
Orbital gyrus	Right	Frontal	1.600	< 0.01
Middle frontal gyrus	Right	Frontal	1.558	< 0.01

**Fig. 2:**
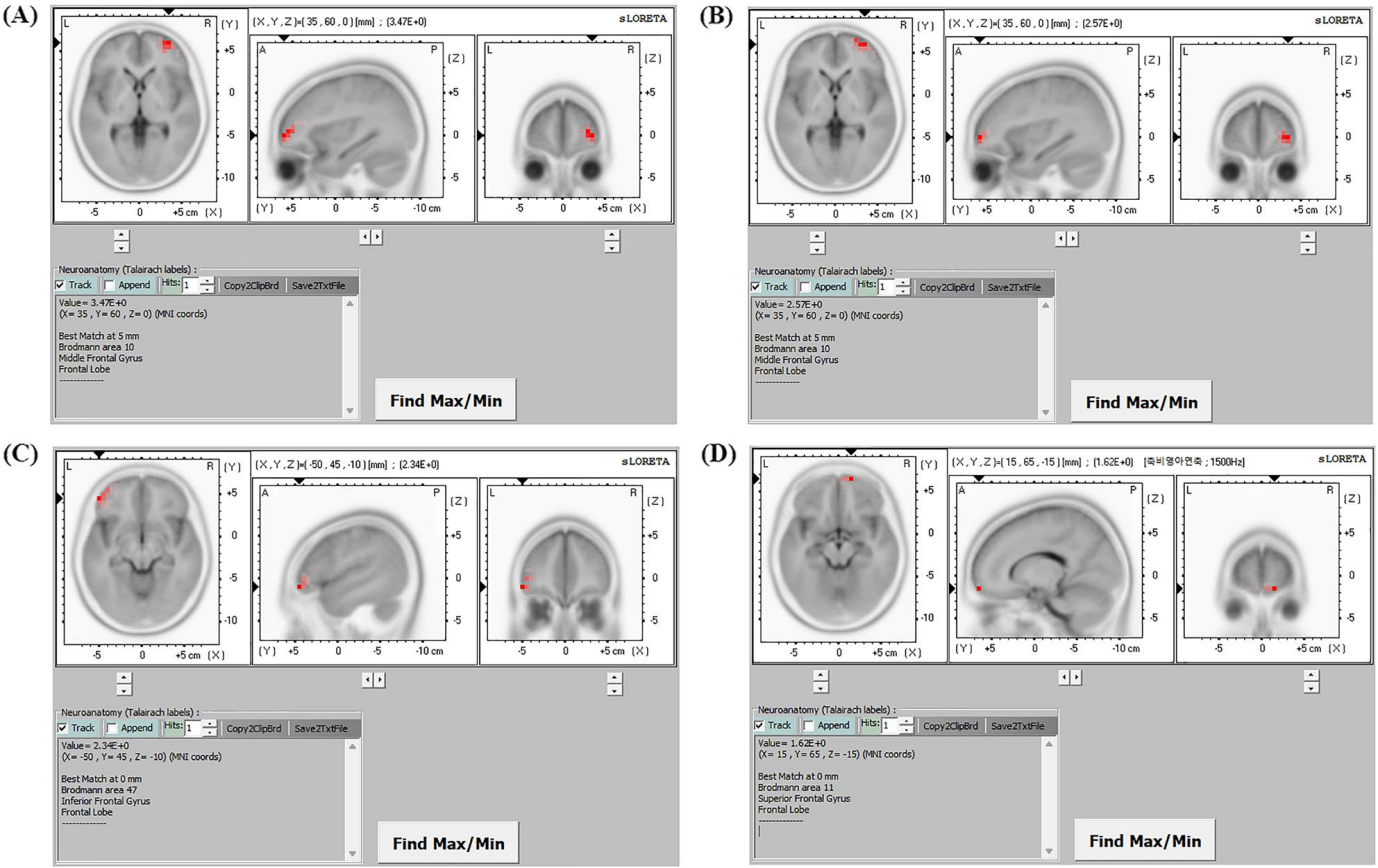
Statistical non-parametric mapping (SnPM) of standardized low-resolution brain electromagnetic tomography (sLORETA) images was projected onto a brain magnetic resonance imaging (MRI) template. Nonparametric statistical analyses were conducted to compare the current density distributions across four frequency bands in the West syndrome group, with comparisons made to the control group. The maximum current density differences were identified in the right middle frontal gyrus for the delta frequency band **A** the right middle frontal gyrus for the theta frequency band **B** the left inferior frontal gyrus for the alpha frequency band **C** and the right superior frontal gyrus for the beta frequency band **D** MNI coord indicates Montreal Neurological Institute coordinate, *A* anterior, *P* posterior, *L* left, *R* right

The most significant difference in current density was identified in the middle frontal gyrus of the right frontal lobe within the delta frequency band (MNI coordinates [x, y, z = 35, 60, 0], Brodmann area 10; log-*F*-ratio = 3.474, *P* < 0.01; Fig. 2a). The next largest difference in current density was also observed within the delta frequency band, encompassing the right superior frontal gyrus (log-*F*-ratio = 3.472, *P* < 0.01), right inferior frontal gyrus (log-*F*-ratio = 3.465, *P* < 0.01), right anterior cingulate of the limbic lobe (log-*F*-ratio = 3.413, *P* < 0.01), and the right insula in the sub-lobar region (log-*F*-ratio = 3.376, *P* < 0.01).

In the theta frequency band, the most significant difference in current density was noted in the right middle frontal gyrus (log-*F*-ratio = 2.573, *P* < 0.01), followed by the right superior frontal gyrus (log-*F*-ratio = 2.572, *P* < 0.01), right inferior frontal gyrus (log-*F*-ratio = 2.562, *P* < 0.01), right medial frontal gyrus (log-*F*-ratio = 2.523, *P* < 0.01), and right anterior cingulate (log-*F*-ratio = 2.479, *P* < 0.01). In the alpha frequency band, the largest difference in current density was observed in the left inferior frontal gyrus (log-*F*-ratio = 2.338, *P* < 0.01), followed by the left middle frontal gyrus (log-*F*-ratio = 2.336, *P* < 0.01), left sub-gyral (log-*F*-ratio = 2.327, *P* < 0.01), left superior frontal gyrus (log-*F*-ratio = 2.306, *P* < 0.01), and left superior temporal gyrus (log-*F*-ratio = 2.283, *P* < 0.01). For the beta frequency band, the most pronounced difference in current density was observed in the right superior frontal gyrus (log-*F*-ratio = 1.616, *P* < 0.01), followed by the right medial frontal gyrus (log-*F*-ratio = 1.610, *P* < 0.01), right rectal gyrus (log-*F*-ratio = 1.602, *P* < 0.01), right orbital gyrus (log-*F*-ratio = 1.600, *P* < 0.01), and right middle frontal gyrus (log-*F*-ratio = 1.558, *P* < 0.01).

## Discussion

This study investigated the electrical neuronal activity in sleep EEGs exhibiting hypsarrhythmia in patients diagnosed with West syndrome and compared these findings with those from healthy control subjects.

The results indicated a significant increase in current density across all frequency bands, with the most pronounced difference observed in the delta band, followed by the theta, alpha, and beta bands. The substantial increase in current density within the delta band aligns with the original characterization of hypsarrhythmia, which is marked by high-amplitude, disorganized slow waves predominantly affecting the delta frequency band, thus corroborating our findings with previous descriptions of the syndrome. Excess activity in the delta band, reflected in our study by elevated current density values, corresponds to increased power in traditional spectral analysis and is typically associated with sustained slow cortical activity, which often indicates dysfunction. However, several recent studies on hypsarrhythmia have reported elevated electrical neuronal activity across various frequency bands, encompassing both low- and high-frequency waves. For example, Burroughs et al.. documented increased absolute power during sleep across all frequency bands (delta, theta, alpha, and beta) in an infantile spasm group compared with controls [4]. Similarly, Smith et al.. observed greater power in the delta, theta, and alpha bands in the waking EEG of an infantile spasm group than in controls, with the beta band exhibiting similar power in the two groups [6]. In another study by Smith et al.., significantly higher EEG power was observed across all frequency bands and channels in sleep EEGs that exhibited hypsarrhythmia [7]. Although not all studies have reported significant differences across all frequency bands, the overall findings are generally consistent with those of the present study.

This study extends the existing body of knowledge by demonstrating a clear gradient of increased current density across all frequency bands in the West syndrome group, with the largest increase in delta and the lowest increase in beta frequencies. The low-to-high frequency gradient observed in this study is an intriguing and distinctive finding, providing novel insights into the dynamics of neuronal activity in West syndrome. This gradient indicates that hypsarrhythmia may be characterized by irregularity and chaos but also by a form of regularity.

Another significant finding of this study was the marked increase in current density, predominantly observed in the frontal lobe, exhibiting an anterior dominant pattern. This pattern was also evident in the three-dimensional cortical models, where an anteroposterior gradient was noted. Previous research on the etiology of West syndrome has produced varied results. In a qEEG study, Smith et al.. reported high broadband amplitude in hypsarrhythmia, particularly in the frontal and central brain regions; however, their study lacked a case-control comparison [6]. Similarly, Haginoya et al.. described a case of a 3-year-old girl with West syndrome who exhibited focal hypsarrhythmia in the right frontal cortex, with interictal SPECT showing hyperperfusion in that region [23]. Sztriha et al.. reported significantly reduced perfusion in the bilateral anterior, mid-frontal, and perisylvian cortex regions, as well as in the left posterior frontal and temporal areas, in a SPECT study comparing patients with West syndrome to controls [24]. Jha et al.. analyzed EEG amplitude progression in 15 patients with hypsarrhythmia and found that high-amplitude discharges originated in the occipital or occipitotemporal areas and spread to adjacent regions [25]. In addition, Japaridze et al.. conducted a coherence analysis of delta activity in hypsarrhythmia and identified the occipital cortex as the primary coherent source [26]. Furthermore, in a SPECT study involving 20 patients with West syndrome, Chiron et al.. reported mixed findings of hyperperfusion and hypoperfusion, predominantly in the frontal and posterior cortices, respectively [27].

These diverse and inconsistent results are difficult to integrate into a cohesive explanation for the origins of hypsarrhythmia in West syndrome. The pathophysiology of West syndrome remains poorly understood, although epileptic spasms and hypsarrhythmia are generally considered to involve subcortical structures. Hypothetically, networks involving both cortical and subcortical structures contribute to the generation of epileptic spasms and hypsarrhythmia in patients with West syndrome [28]. While sLORETA primarily focuses on cortical activity, several multimodal studies have provided complementary evidence highlighting the involvement of subcortical structures. Maki et al. used simultaneous EEG–functional magnetic resonance imaging (fMRI) and demonstrated that positive blood oxygen level-dependent (BOLD) responses related to hypsarrhythmia appeared in the thalamus, hippocampus, and brainstem [29]. Similarly, Siniatchkin et al. reported that slow wave activity in hypsarrhythmia correlated significantly with BOLD signals in the brainstem and thalamus, as well as in cortical regions, predominantly in the frontal and parietal cortices [30]. In a subsequent connectivity analysis using the directed coherence method, it was further shown that in West syndrome, the strongest and most significant information flow was directed ascendingly from the brainstem toward the basal ganglia and cerebral cortex, supporting the theory that hypsarrhythmia results from ascending brainstem pathways that widely project to basal ganglia and cortical regions [26].

Based on these findings, the results potentially reflect an anteroposterior gradient within these cortical-subcortical networks rather than a specific origin within a particular region [31]. The anteroposterior gradient in the current density observed in hypsarrhythmia is another distinctive finding of the present study. The importance of this study is the suggestion of potential involvement and dysfunction of the anterior regions, particularly the frontal lobe, in West syndrome.

The results of this study offer novel information regarding hypsarrhythmia associated with West syndrome, emphasizing the presence of a low-to-high and anteroposterior gradient in neuronal activity. Notably, this is the first study in which the source of hypsarrhythmia was localized using sLORETA in the context of West syndrome, contributing to the understanding of electrophysiological characteristics. The findings suggest that hypsarrhythmia, traditionally characterized by a chaotic and irregular nature, may also exhibit underlying frequency and spatial organization, with the anterior and frontal cortices playing a significant role in its manifestation. This challenges conventional perspectives and emphasizes the need for a more nuanced interpretation of hypsarrhythmic patterns.

However, the study has several limitations. First, the relatively small sample size limits the generalizability of the findings. Future studies with larger cohorts are necessary to enhance statistical power and validate the results across a broader population. In addition, although sLORETA is a valuable tool for cortical source localization without location bias, analysis is confined to the cortical gray matter, hippocampus, and amygdala. Consequently, deep subcortical structures, such as the thalamus and brainstem, which are likely involved in West syndrome pathology, were not assessed in this study. To overcome this limitation, other advanced neuroimaging techniques should be considered in future investigations. Despite these limitations, the SnPM method using built-in voxel-wise randomization tests with 5,000 permutations was well-suited for the sample size and provided robust statistical results [16, 17]. However, a larger sample size and use of alternative analytical approaches would be beneficial to confirm and refine the findings.

This study contributes to the growing body of knowledge on West syndrome by using EEG source localization with sLORETA. However, it is important to acknowledge that West syndrome is etiologically and clinically heterogeneous, with patients exhibiting a range of structural, genetic, metabolic, and unknown causes, as well as varying symptom severity. This heterogeneity could influence the EEG findings and limit the generalizability of our results. Therefore, future research should include larger, stratified cohorts to better understand how these factors affect EEG patterns.

While sLORETA provides valuable cortical localization, it cannot assess deep subcortical structures, which may play a crucial role in the pathology of West syndrome. To address this limitation, future studies should incorporate neuroimaging techniques such as fMRI, positron emission tomography (PET), and single-photon emission computed tomography (SPECT) to evaluate deep subcortical regions. These methods would provide important insights into the subcortical contributions to the disorder, complementing the cortical data obtained through sLORETA. Moreover, incorporating simultaneous EEG-fMRI studies, magnetoencephalography (MEG), and advanced connectivity analyses will allow for a more integrated understanding of brain activity, combining cortical and subcortical perspectives, as well as providing deeper insights into the functional connectivity and dynamic neural networks involved in hypsarrhythmia.

Additionally, while this study focuses on comparing EEG patterns between the West syndrome and control groups at the time of diagnosis, we recognize the potential clinical value of exploring correlations between EEG findings and developmental outcomes or treatment responses. Future research, particularly longitudinal studies, will be valuable in investigating whether EEG findings, such as stronger delta activity or more anterior predominance, are associated with developmental outcomes or treatment responses. These clinical correlations, when integrated with advanced neuroimaging techniques, would be crucial to deepen our understanding of the neural mechanisms underlying West syndrome.

Ultimately, an integrated approach combining advanced neuroimaging methods with qEEG is essential to refine our understanding of the pathophysiology of West syndrome. Future studies should aim for larger, stratified cohorts and multimodal imaging to provide a more holistic understanding of both cortical and subcortical contributions to the disorder.

## Conclusion

Our study reveals a significant increase in current density across all frequency bands in West syndrome, with the most pronounced differences in the delta band. These changes exhibited a predominant anterior distribution, particularly in the frontal lobe. The observed low-to-high and anteroposterior gradients in neuronal activity offer new insights into the hypsarrhythmia associated with West syndrome. These findings contribute to the expanding body of research on West syndrome and emphasize the significance of qEEG analysis in enhancing the interpretation of hypsarrhythmia. Future research incorporating multimodal neuroimaging and larger patient cohorts are needed to further validate these findings and advance the understanding of this complex DEE.

## Data Availability

The datasets analyzed during the current study are available from the corresponding author upon reasonable request.

## Abbreviations

EEG: electroencephalography
DEE: developmental epileptic encephalopathy
qEEG: quantitative electroencephalography
FFT: fast Fourier transform
sLORETA: standardized low-resolution brain electromagnetic tomography
MNI: Montreal Neurological Institute
SnPM: statistical non-parametric mapping
SPECT: single-photon emission computed tomography
